# NCI 159456 PERK Inhibitor as a Targeted Therapy for Lung Cancer: An In Vitro Study

**DOI:** 10.3390/biomedicines12040889

**Published:** 2024-04-17

**Authors:** Wioletta Rozpędek-Kamińska, Grzegorz Galita, Natalia Siwecka, Zuzanna Granek, Julia Barczuk, Kamil Saramowicz, Ireneusz Majsterek

**Affiliations:** Department of Clinical Chemistry and Biochemistry, Medical University of Lodz, 90-419 Lodz, Poland; wioletta.rozpedek@umed.lodz.pl (W.R.-K.); grzegorz.galita@umed.lodz.pl (G.G.); natalia.siwecka@stud.umed.lodz.pl (N.S.); zuzanna.granek@stud.umed.lodz.pl (Z.G.); julia.barczuk@stud.umed.lodz.pl (J.B.); kamil.saramowicz@stud.umed.lodz.pl (K.S.)

**Keywords:** ER stress, UPR, PERK, apoptosis, lung cancer, PERK inhibitor, lung cancer treatment

## Abstract

Non-small cell lung cancer (NSCLC) represents the most common histological type of lung cancer, characterized by a five-year survival rate of 15% and poor prognosis. Accumulating evidence indicates a prominent role of endoplasmic reticulum (ER) stress and the protein kinase RNA-like ER kinase (PERK)-dependent pathway of the unfolded protein response (UPR) in the pathogenesis of NSCLC. Increased expression of downstream targets of PERK was observed in various subtypes of NSCLC, and it was associated with a more aggressive phenotype, high risk of recurrence, and poor prognosis. Therefore, the present study aimed to investigate the biological effect of the selective PERK inhibitor NCI 159456 on A549 NSCLC cells and Human Pulmonary Fibroblasts (HPF) in vitro. Treatment of both normal and ER-stressed A549 cells with NCI 159456 resulted in a significant increase in the mRNA expression level of pro-apoptotic genes like *activating transcription factor 4* (*ATF4)*, *DNA damage inducible transcript 3* (*DDIT3)*, and *BCL2 Associated X, Apoptosis Regulator* (*BAX)* as well as a decreased level of the anti-apoptotic gene *B-cell lymphoma 2 (Bcl-2)*. Cytotoxicity and genotoxicity analyses revealed that NCI 159456 significantly decreased viability and increased DNA damage in A549 cells under normal and ER stress conditions. Caspase-3 and reactive oxygen species (ROS) detection assays demonstrated that NCI 159456 significantly induced apoptosis and increased the ROS level in normal and ER-stressed A549 cells. Importantly, treatment with the inhibitor did not affect substantially normal HPF cells at any used concentration. The results indicate that PERK inhibitors could potentially be applied as a targeted therapy for NSCLC.

## 1. Introduction

Lung cancer is the most common cause of cancer-related mortality worldwide, accounting for approximately 1.8 million deaths per year [1]. Non-small cell lung cancer (NSCLC) represents about 84% of all lung cancers, of which adenocarcinoma (54.7%) and squamous cell carcinoma (29.4%) are the most prevalent histological subtypes [2]. NSCLCs are a heterogeneous group of tumors initiated by many driver mutations [3]. Oncogenic mutations disrupt cell cycle control, leading to high proliferation and rapid growth, exacerbating the cell’s translational and metabolic demands. During progression, cancer cells are exposed to multiple cell-extrinsic stressors like hypoxia, nutrient deprivation, acidosis, and oxidative stress [4]. These factors interfere with the capability of the endoplasmic reticulum (ER) machinery to fold, assemble, and transport newly synthesized proteins. Consequently, unfolded proteins accumulate within the ER lumen, causing ER stress [5]. To alleviate stress and restore proteostasis, ER activates the adaptive signaling pathways known as the unfolded protein response (UPR). The first-line adaptive response to ER stress is initiated by protein kinase R-like ER kinase (PERK), which phosphorylates its major substrate eukaryotic initiation factor 2α (eIF2α) [6]. This results in global attenuation of protein translation with preferentially enhanced expression of the activating transcription factor 4 (ATF4). ATF4 upregulates the expression of adaptive UPR target genes implicated in antioxidant response, amino acid metabolism, and cytoprotective autophagy to maintain cancer cell survival and growth. Although the PERK pathway activates pro-apoptotic factors like *DNA damage inducible transcript 3* (*DDIT3*) and *B-cell lymphoma-2* (*Bcl-2*) family proteins, recent reports suggest that in cancer cells the balance is shifted toward pro-survival signaling [7,8]. PERK-mediated pathway promotes cancer cell survival and actively contributes to carcinogenesis by interacting with several pro-oncogenic signaling pathways, including c-Myc, Wnt, and PI3K/AKT [9,10,11,12]. Furthermore, activation of the PERK-ATF4 signaling pathway promotes metastasis and confers resistance to chemotherapy and radiotherapy, representing one of the most critical challenges for cancer treatment [13,14,15]. Therefore, PERK emerges as a promising target for therapeutic intervention in multiple malignancies [16]. Interestingly, recent studies suggest that PERK-specific inhibitors may target dormant tumor cells resistant to conventional antiproliferative therapies, thus providing an adjuvant approach to eliminate minimal residual disease and prevent recurrence [17]. While many PERK inhibitors have been investigated as potential anti-cancer agents [18,19,20], there are few reports on their applicability in NSCLC treatment. ER stress, followed by the activation of the PERK-dependent pathway, is a common phenomenon in NSCLCs, and elevated levels of PERK downstream targets are observed across various NSCLC histological subtypes [21,22,23,24]. In human lung adenocarcinomas, increased expression of both p-eIF2α and ATF4 is mainly observed in advanced tumor subtypes with aggressive growth patterns, high risk of recurrence, and poor prognosis [22,23]. Furthermore, cigarette smoking, a major etiologic factor of NSCLC, has been shown to induce PERK-dependent eIF2α phosphorylation in the human pulmonary epithelium, indicating the potential involvement of the PERK pathway in NSCLC development [24]. ATF4-mediated protection against reactive oxygen species (ROS) has also been linked to radio- and chemotherapy resistance in various cancer cells, including those derived from NSCLCs [25]. All in all, these findings demonstrate that activation of the PERK-related pathway may contribute to the development and progression of NSCLC, supporting the resistance of tumor cells to metabolic limitations and treatment.

Considering the implications of PERK activation in human NSCLCs tumorigenesis, in this study, we assessed the properties of the small-molecule PERK inhibitor NCI 159456 in a cellular model of NSCLC. NCI 159456 was selected from the National Institute of Cancer (NCI) by a high-throughput inhibitor screening assay for its low rate of compounds containing known toxicophores and reactive functional groups and its maximum molecular diversity. NCI 159456 is a compound with a molecular mass of 338 Da, which has shown marked cytotoxicity against breast cancer lines in a previous study [26].

## 2. Materials and Methods

### 2.1. Screening for the NCI 159456 PERK Inhibitor

High-throughput assay (HTS) was used to select the NCI 159456 PERK inhibitor from the National Cancer Institute (NCI) compound library. The PERK inhibitory compounds were selected based on the following properties: low rate of compounds that contain known toxicophores or reactive functional groups and maximization of molecular diversity. A time-resolved fluorescence resonance energy transfer (TR-FRET) assay was used to screen a small-molecule NCI compound library.

### 2.2. Cell Cultures

The experiments were carried out using commercially available human lung carcinoma epithelial cells (A549; CRM-CCL-185) obtained from the American Type Culture Collection (ATCC; Manassas, VA, USA) and Human Pulmonary Fibroblasts (HPF; 3300) obtained from the ScienCell Research Laboratories (Carlsbad, CA, USA). Both cell cultures were maintained under standard conditions (37 °C; 5% CO_2_; 95% humidity), as described by the manufacturer’s guidelines. The A549 cell line was cultured in Kaighn’s Modification of Ham’s F-12 Medium (F-12K; ATCC; USA) with supplementation of 10% (*v*/*v*) fetal bovine serum (FBS; Sigma-Aldrich Corp., St. Louis, MO, USA), 100 U/mL penicillin, and 100 μg/mL streptomycin solution (P/S; GIBCO-BRL, Life Technologies, Ltd., Renfrew, UK). A549 cells were cultured in T-75 culture vessels and passaged every 2–3 days at 90–95% confluency after dissociation with 0.25% (*w*/*v*) Trypsin-0.53 mM EDTA (T/E) solution (ATCC; USA). The HPF cell line was cultured in the Fibroblast Medium (FM; ScienCell Research Laboratories, USA) containing fibroblast basal medium (BM) supplemented with 2% fetal bovine serum (FBS), 1% fibroblast growth supplement, and 1% P/S antibiotic solution. HPF cells were cultured in poly-L-lysine-coated T-75 culture flasks (2 μg/cm^2^). HPF cells were split every 3–4 days at 90–95% confluency and after detachment with 0.05% T/E solution (ScienCell Research Laboratories, USA).

### 2.3. Gene Expression Analysis

To determine the mRNA expression levels of the pro-apoptotic genes linked to ER stress, total RNA was extracted from A549 and HPF cells utilizing PureLink RNA Mini Kit (Thermo Fisher Scientific Inc., Waltham, MA, USA) in accordance with the manufacturer’s protocol. Subsequently, the obtained RNA was transcribed to cDNA via the GoScript™ Reverse Transcriptase (Promega Inc., Madison, WI, USA; final concentration 100 ng). Next, to assess the expression profile of pro-apoptotic genes related to ER stress, namely *DDIT3* (Hs01090850_m1), *BCL2 Associated X, Apoptosis Regulator* (*BAX)* (Hs00180269_m1), *ATF4* (Hs00909569_g1), and *Bcl-2* (Hs00608023_m1), the TaqMan Gene Expression Assay was used. *ACTB* (Hs99999903_m1) served as a reference gene. The qPCR analysis mixture (total volume of 10 μL) consisted of cDNA (1 μL), primers (1 μL), 5× HOT FIREPol^®^ Probe quantitative (q)PCR Mix (2 μL; Solis BioDyne OÜ, Tartu, Estonia), and nuclease-free water (6 μL). The conditions for the qPCR reaction were set according to the manufacturer’s manual: 15 min enzyme activation at 95 °C and 40 cycles of denaturation (10 s, 95 °C) with annealing/extension (60 s, 60 °C). The mRNA expression level was determined via the Bio-Rad CFX96 (BioRad Laboratories, Hercules, CA, USA) system.

### 2.4. Cytotoxicity Analysis

The cytotoxicity assessment of the evaluated NCI 159456 PERK inhibitor was performed via the 2,3-bis-(2-methoxy-4-nitro-5-sulfophenyl)-2*H*-tetrazolium-5-carboxanilide (XTT) colorimetric assay (Thermo Scientific). The assay measures cellular metabolic activity, indicating cell viability, proliferation, and cytotoxicity. XTT colorimetric assay is based on reducing a yellow tetrazolium salt to an orange formazan dye by living, metabolically active cells. All experiments were carried out three times with similar results. A549 and HPF cells were seeded in a 96-well plate (5 × 10^3^/well) and cultured for 24 h in 100 μL of the complete F-12K or FM growth medium, respectively. Subsequently, A549 and HPF cells were treated with 100 μL of the complete medium containing NCI 159456 compound at a wide concentration range (3 μM, 6 μM, 12 μM, 25 μM, 50 μM, 75 μM, 100 μM, 50 mM) or the solvent for the evaluated PERK inhibitor, 0.1% DMSO (Sigma-Aldrich Corp.). The positive controls constituted cells incubated with 100% DMSO, whereas the negative control cells were grown only in the complete F-12K or FM medium, respectively. Then, the effect of the NCI 159456 PERK inhibitor in ER-stressed A549 cells was analyzed. The A549 cells were seeded in 96-well plates (5 × 10^3^/well) in 100 μL of the complete F-12K growth medium and left for 24 h to adhere. Next, A549 cells were preincubated with 100 μL of the complete medium containing NCI 159456 PERK inhibitor at 3 μM and 50 μM concentrations for 1 h. After incubation, the A549 cells were treated with Th (500 nM), an ER stress inducer. Some cells were exposed only to 500 nM Th. The untreated A549 cells grown in the complete medium served as the negative control, and the positive control constituted cells treated with 100% DMSO. All samples were incubated for 16, 24, or 48 h with the respective compounds or media. Then, 25 µL of XTT/PMS suspension was added to each well in accordance with the manufacturer’s protocol. After 2 h of incubation in a 5% CO_2_ incubator at 37 °C, the absorbance measurement was performed using the Synergy HT spectrophotometer (BioTek, Shoreline, WA, USA) at a wavelength of 450 nm.

### 2.5. Genotoxicity Analysis

The comet assay, alkaline version (ACA) was used to study the genotoxic impact of the investigated NCI 159456 PERK inhibitor on A549 and HPF cells. All experiments were run in triplicate with similar results. A549 and HPF cells were seeded at 2 × 10^5^ density in 6-well plates in 2 mL of F-12K or FM growth medium, respectively. Subsequently, A549 and HPF cells were exposed to the NCI 159456 PERK inhibitor at a wide range of concentrations (3–100 μM) or the solvent, 0.1% DMSO (Sigma-Aldrich Corp.), for 24 h. Cells treated with 5% DMSO (Sigma-Aldrich Corp.) were used as the positive control, and cells cultured only in the complete F-12K or FM growth medium, respectively, constituted the negative control. Furthermore, the genotoxic effect of the NCI 159456 PERK inhibitor was analyzed in Th-treated A549 cells. A549 cells were seeded in 6-well plates at 2 × 10^5^ in 2 mL of the complete F-12K cell culture medium for 24 h. Next, the A549 cells were preincubated for 1 h with 2 mL of the complete medium with the NCI 159456 PERK inhibitor at 3 μM and 50 μM concentrations. Following this, A549 cells were exposed to 50 nM Th. Some of the cells were incubated only with 50 nM Th. The positive control constituted A549 cells exposed to 5% DMSO (Sigma-Aldrich Corp.), whereas A549 cells incubated in 2 mL of the complete medium served as the negative control. All the treated samples were incubated for 24 h. The cells were then suspended in 0.37% low melting point agarose and placed on microscope slides pre-coated with normal melting point agarose (Sigma-Aldrich Corp.). The samples were incubated for 1 h at 4 °C in pH 10 lysis buffer (2.5 M NaCl, 10 mM Tris, 100 mM EDTA, 1% TritonX-100) (Sigma-Aldrich Corp.). Next, the preparations were incubated for 20 min at 4 °C in the development buffer (300 mM NaOH, 1 mM EDTA) and submitted to electrophoresis in the electrophoretic buffer (30 mM NaOH, 1 mM EDTA) for 20 min at 4 °C (32 mA, 17 V). After electrophoresis, the preparations were washed 3 times with distilled water and left to dry at room temperature. Then, the samples were stained with a fluorescent dye, DAPI. Cell DNA damage was analyzed under a fluorescence microscope by measuring the DNA percentage in the comet tail.

### 2.6. Apoptosis Analysis

The caspase-3 level in A549 and HPF cells was analyzed using the Caspase-3 Assay Kit (Colorimetric; Abcam, Cambridge, UK). Caspase-3 acts as an executioner caspase during apoptosis, as its essential proteolytic functions lead to the final stages of programmed cell death. All experiments were run in triplicate with similar results. A549 and HPF cells were seeded in 6-well plates at 5 × 10^5^/well in the complete F-12K or FM growth medium and left to adhere for 24 h. Next, A549 and HPF were incubated with the tested NCI 159456 PERK inhibitor at 3 to 100 μM concentrations or with the solvent 0.1% DMSO (Sigma-Aldrich Corp.) for 24 h. Cells incubated for 16 h with 1 µM of staurosporine (Sigma-Aldrich Corp.) served as the positive control. In contrast, cells incubated for 24 h in F-12K or FM growth medium constituted the negative control. Moreover, to assess the effect of NCI 159456 compound on Th-treated A549 cells, the cells were seeded at 5 × 10^5^/well in 6-well plates and grown for 24 h in the complete medium. Next, A549 cells were treated with the complete medium containing the NCI 159456 PERK inhibitor at 3 μM and 50 μM concentrations for 1 h before exposure to 500 nM Th for 24 h. Some A549 cells were exposed only to Th at 500 nM for 24 h. The positive controls constituted cells exposed to 16 h treatment with 1 µM staurosporine (Sigma-Aldrich Corp.), and the negative controls constituted cells cultured for 24 h in the complete A549 growth medium only. Subsequently, A549 cells were rinsed once with 1× DPBS (Sigma-Aldrich Corp.) and dissociated with 0.25% T/E solution (Innoprot, Derio, Spain). The resulting cell suspension was centrifuged at room temperature for 5 min at 1000 rpm, and then, the supernatant was removed. The pellet was then resuspended in the complete F-12K medium; the cells were counted and centrifuged at room temperature for 5 min at 1000 rpm. The pellet (1 × 10^6^ cells) was resuspended in the cold Cell Lysis Buffer (50 µL) and incubated on ice for 10 min. Next, the samples were centrifuged for 1 min at 10,000× *g*. The obtained supernatants were transferred to fresh 2 mL tubes. The protein concentration was calculated and normalized to 100 μg protein/sample by performing a standard Bradford assay, in which BSA was used as a protein standard. Subsequently, 2× Reaction Buffer (10 mM DTT, 4 mM DEVD-pNA) was added to each sample, and the samples were incubated at 37 °C for 2 h. Following the incubation, the p-NA absorbance was measured by the Synergy HT spectrophotometer (BioTek, USA) at 405 nm wavelength.

### 2.7. Evaluation of the Level of Reactive Oxygen Species (ROS)

The evaluation of the level of reactive oxygen species (ROS) was performed using the Reactive Oxygen Species (ROS) Detection Assay Kit (Abcam, Cambridge, UK). A549 and HPF cells were grown in 96-well plates (6 × 10^4^/well) for 24 h in 100 μL of the complete F-12K or FM growth medium, respectively. Cells grown only in the complete F-12K or FM growth medium, respectively, constituted the negative control, whereas cells incubated with ROS inducer constituted the positive control. After the cells’ adhesion, the cell culture medium was removed, and all wells were rinsed with 100 µL of ROS Assay buffer. Subsequently, the ROS Label was added to each well. After 45 min of incubation in the dark at 37 °C, the ROS Label was removed and A549 and HPF were exposed for 24 h to 100 μL of the complete culture medium containing NCI 159456 compound at a wide concentrations range (3–100 μM) or 0.1% DMSO (Sigma-Aldrich Corp.) that was used as the solvent for the evaluated PERK inhibitor. Then, the culture medium with the test compounds was removed, and ROS Assay buffer (100 µL) was added to each well. The fluorescence was measured at Ex/Em = 495/529 nm by a Synergy HT spectrophotometer (BioTek, USA).

### 2.8. Statistical Analysis

The statistical analysis was carried out by the Statistica (version 13; StatSoft, Kraków, Poland). In the present study, the Shapiro–Wilk test was applied to determine the normality of data distribution in all experiments. Except for the comet assay, all data were characterized by a normal distribution. Therefore, further analysis and comparison between multiple groups were performed by ANOVA with Dunnett’s post hoc test. In the comet assay, the two groups were compared using the Mann–Whitney *U*-test. The statistical analysis in each experiment was based on the results of three replicates. In the graphs, statistically significant differences are marked as follows: * *p* < 0.05, ** *p* < 0.01, *** *p* < 0.001.

## 3. Results

### 3.1. mRNA Expression Analysis of the ER Stress-Related Pro-Apoptotic Genes in A549 and HPF Cells Treated with NCI 159456 PERK Inhibitor

**Figure 1:**
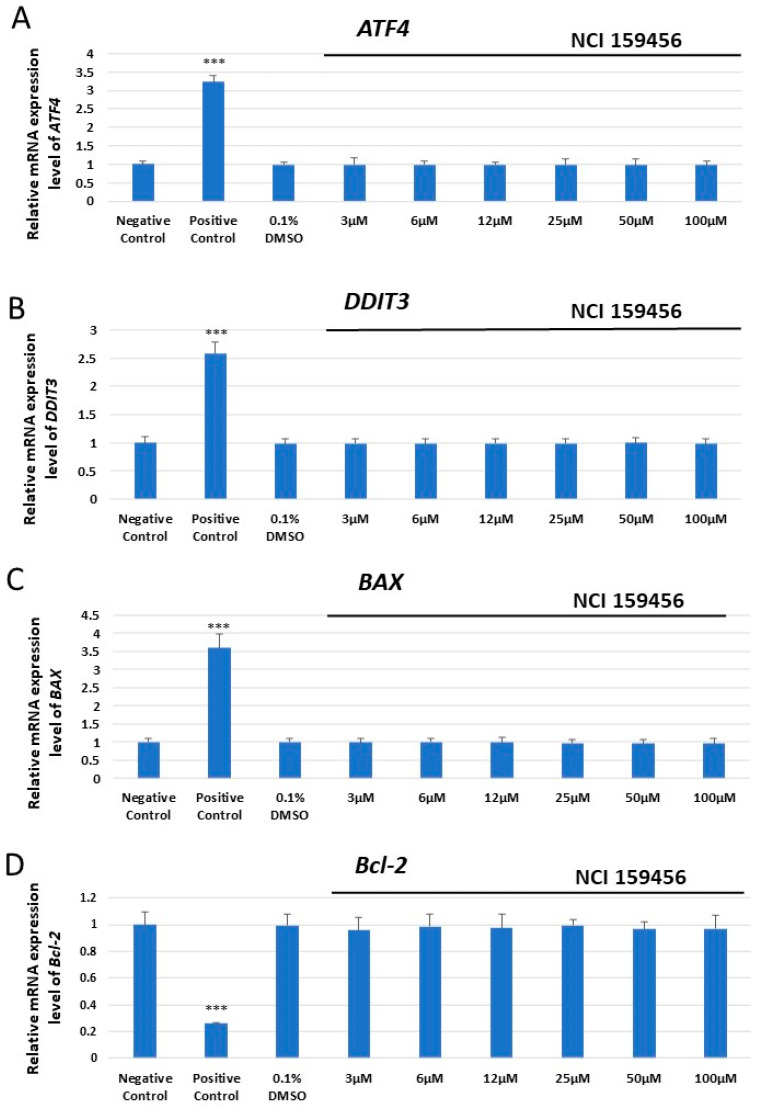
Evaluation of the mRNA expression level of the apoptotic ER stress-related genes: *ATF4* (**A**), *DDIT3* (**B**), *BAX* (**C**), and *Bcl-2* (**D**) in HPF cells exposed to the NCI 159456 PERK inhibitor. The TaqMan gene expression assay was performed for the analysis. All experiments were run in triplicate; values are expressed as mean ± SEM, n = 3; *** *p* < 0.001 versus the negative control; Negative Control—untreated HPF cells; Positive Control—100 µM H_2_O_2_ (37 °C, 24 h); 0.1% DMSO—HPF cells treated with the solvent, 0.1% dimethyl sulfoxide.

**Figure 2:**
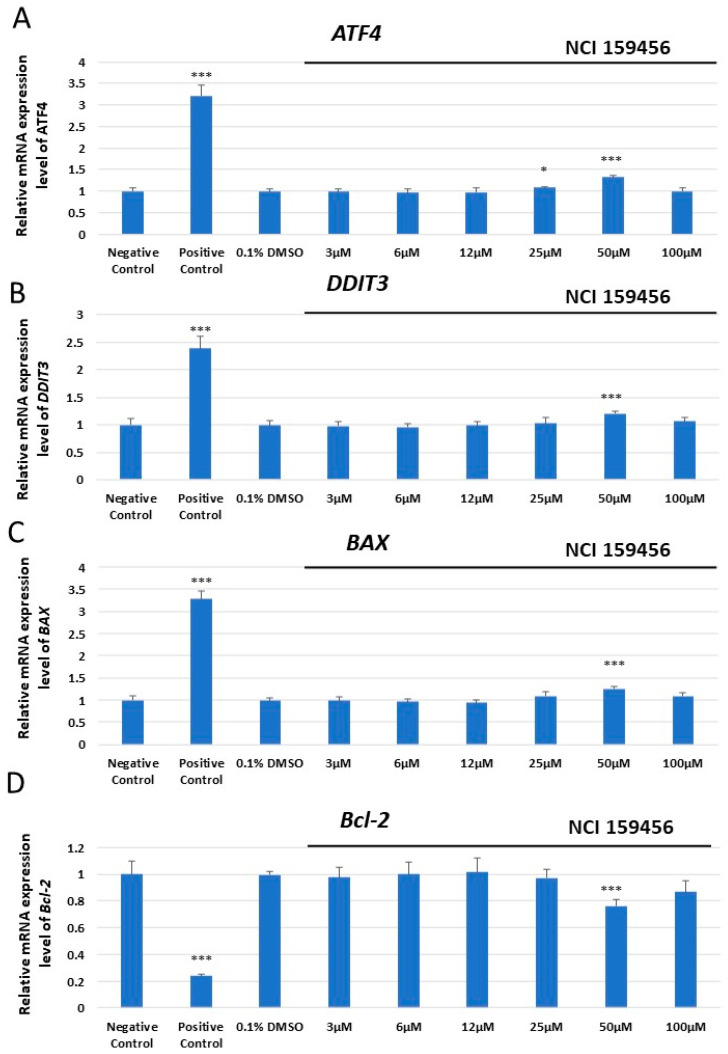
Evaluation of the mRNA expression level of the apoptotic ER stress-related genes: *ATF4* (**A**), *DDIT3* (**B**), *BAX* (**C**), and *Bcl-2* (**D**) in A549 cells exposed to the NCI 159456 PERK inhibitor. The TaqMan gene expression assay was performed for the analysis. All experiments were run in triplicate; values are expressed as mean ± SEM, n = 3. * *p* < 0.05, *** *p* < 0.001 versus the negative control. Negative Control—untreated A549 cells; Positive Control—100 µM H_2_O_2_ (37 °C, 24 h); 0.1% DMSO—A549 cells treated with the solvent, 0.1% dimethyl sulfoxide.

**Figure 3:**
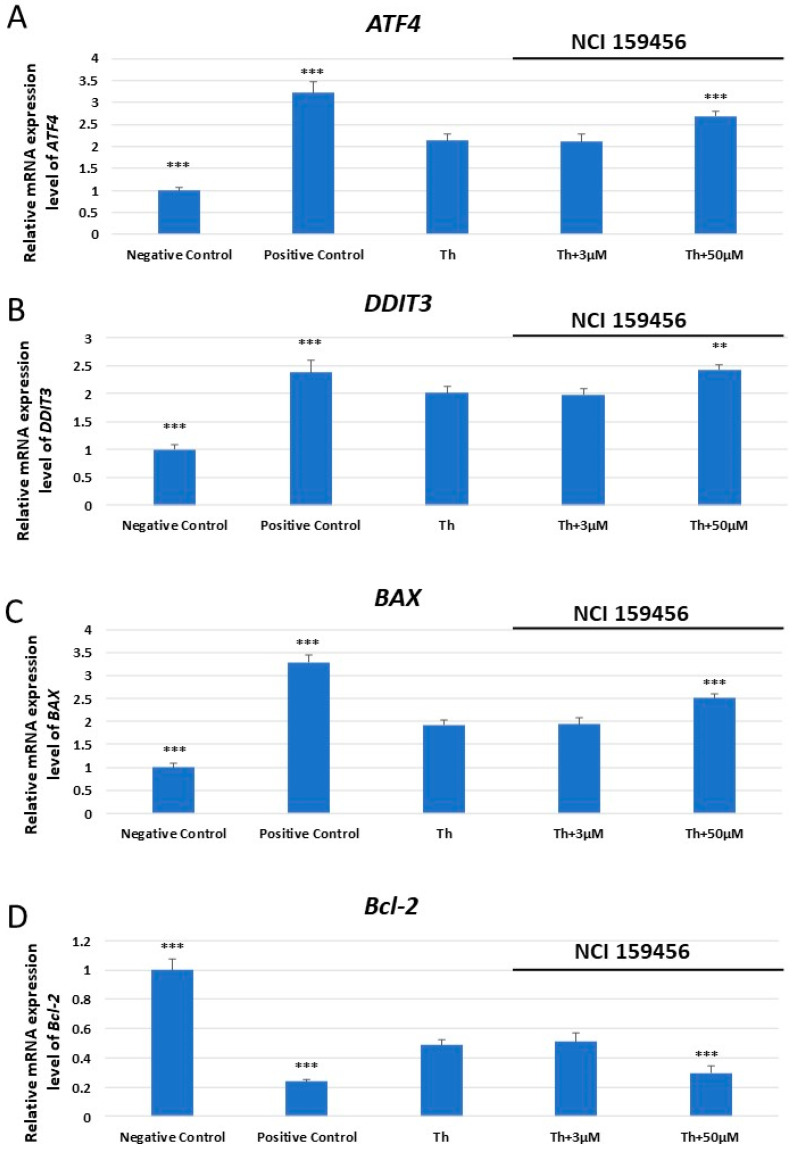
Evaluation of the mRNA expression level of the apoptotic ER stress-related genes: *ATF4* (**A**), *DDIT3* (**B**), *BAX* (**C**), and *Bcl-2* (**D**) in A549 cells exposed to Th alone or Th and NCI 159456 PERK inhibitor. The TaqMan gene expression assay was performed for the analysis. All experiments were run in triplicate; values are expressed as mean ± SEM, n = 3. ** *p* < 0.01, *** *p* < 0.001 versus Th. Negative Control—untreated A549 cells; Positive Control—100 µM H_2_O_2_ (37 °C, 24 h); Th—thapsigargin-treated A549 cells (ER-stressed A549).

The expression analysis of *ATF4*, *DDIT3* encoding DNA damage-inducible transcript 3 (CHOP), *BAX*, and *Bcl-2* mRNA was performed both in HPF and A549 cells exposed to the investigated NCI 159456 compound (3–100 μM) or with 0.1% DMSO, as well as in cancer A549 cells under Th-induced ER stress and incubated with the NCI 159456 PERK inhibitor (3 and 50 μM). No significant changes in *ATF4*, *DDIT3*, *BAX*, and *Bcl-2* mRNA expression were observed in non-cancerous HPF cells at any investigated NCI 159456 compound concentration and 0.1% DMSO compared to the negative control (Figure 1). Interestingly, obtained results have shown a significant increase in the mRNA expression level of the *ATF4*, *DDIT3*, and *BAX* pro-apoptotic genes, as well as a significant decrease in the level of anti-apoptotic gene *Bcl-2* in cancer A549 cells treated with 50 μM of the NCI 159456 PERK inhibitor compared to that in the negative control cells. No significant changes were detected in ATF4, DDIT3, BAX, and Bcl-2 mRNA expression levels in A549 cells after incubation with 0.1% DMSO compared to that in the negative control cells (Figure 2). Further, A549 cells that were incubated both with Th and 50 μM NCI 159456 PERK inhibitor showed a significant increase in the expression level of pro-apoptotic, ER stress-related genes *ATF4*, *DDIT3*, and *BAX* compared with that in A549 cells exposed to Th alone. A549 cells with Th-induced ER stress conditions treated with 50 μM NCI 159456 compound showed significantly lower expression level of the anti-apoptotic gene *Bcl-2* than that in the untreated ER-stressed A549 cells (Figure 3).

### 3.2. Evaluation of the Cellular Toxicity of the NCI 159456 PERK Inhibitor

**Figure 4:**
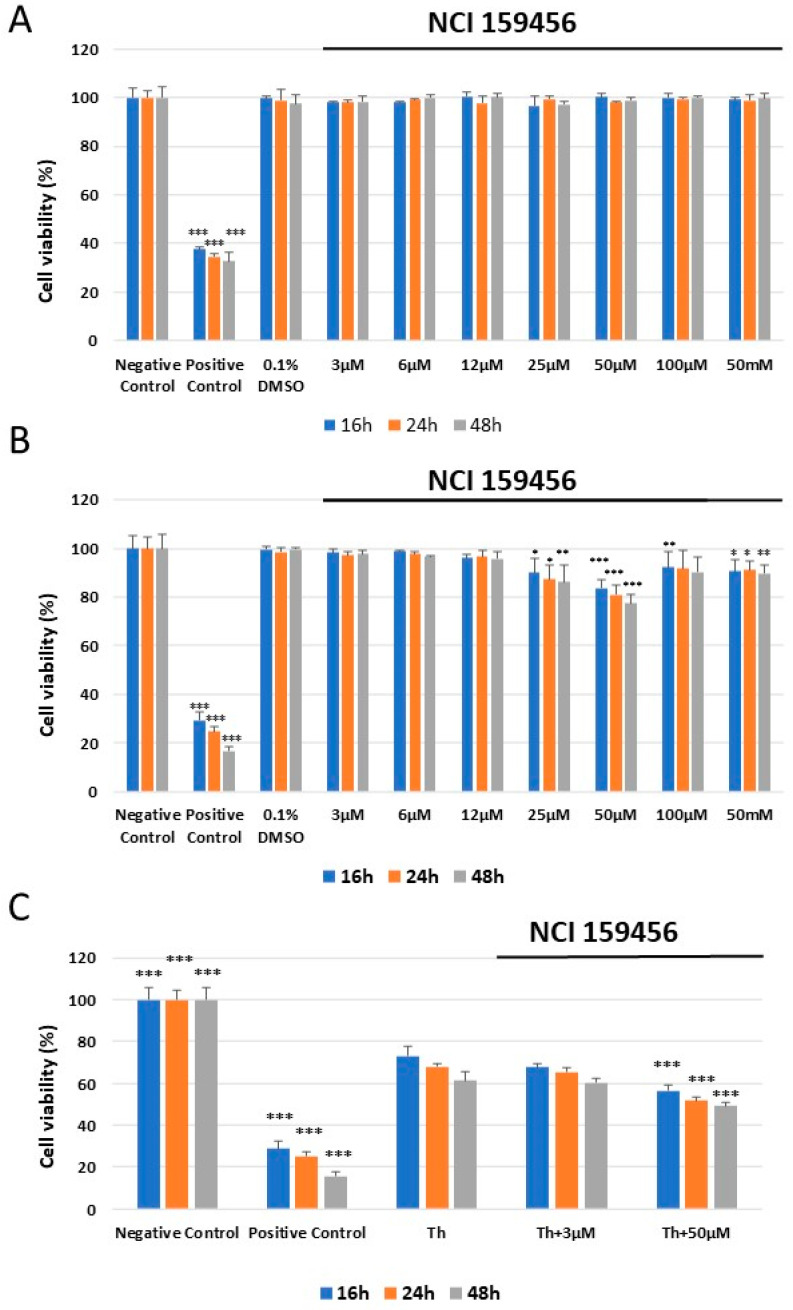
Analysis of the cytotoxicity of the NCI 159456 PERK inhibitor toward HPF cells (**A**), A540 cells (**B**), and viability of A549 cells exposed to Th alone and ER-stressed A549 cells treated with the NCI 159456 PERK inhibitor (**C**) performed by the XTT assay. All experiments were run in triplicate; values are expressed as mean ± SEM, n = 3. * *p* < 0.05, ** *p* < 0.01, *** *p* < 0.001 versus the negative control (**A**,**B**) and Th (**C**). Negative Control—untreated HPF (**A**) and A549 (**B**,**C**) cells; Positive Control—HPF (**A**) and A549 (**B**,**C**) cells treated with 100% dimethyl sulfoxide; 0.1% DMSO—HPF (**A**) and A549 (**B**) cells treated with the solvent, 0.1% dimethyl sulfoxide; Th—thapsigargin-treated A549 cells (ER-stressed A549 cells).

The cytotoxic effect of the investigated NCI 159456 compound both in HPF and A549 cell lines was evaluated via the XTT colorimetric assay. No significant cytotoxicity was noted toward the HPF cell line at any applied concentrations of the tested NCI 159456 PERK inhibitor or 0.1% DMSO after 16, 24, or 48 h compared with the negative control (Figure 4A). However, the obtained results demonstrated a significantly reduced percentage of viable A549 cells following the 16, 24, or 48 h incubation with 50 μM of NCI 159456 PERK inhibitor compared with the negative control. Also, 0.1% DMSO did not induce significant toxicity in A549 cells after the 16, 24, or 48 h incubation compared to the negative control (Figure 4B). Moreover, the results showed a significant decrease in the viability of A549 cells with Th-induced ER stress upon treatment with 50 μM of NCI 159456 compound compared to the untreated ER-stressed A549 cells at each incubation time (Figure 4C).

### 3.3. Genotoxicity Assessment of the NCI 159456 PERK Inhibitor

**Figure 5:**
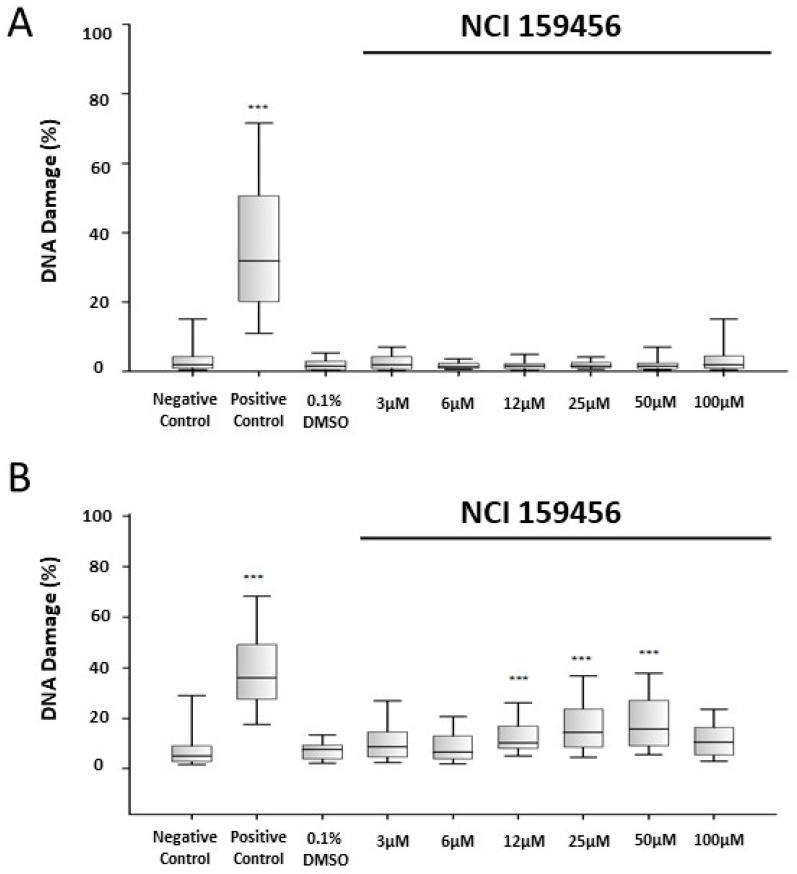

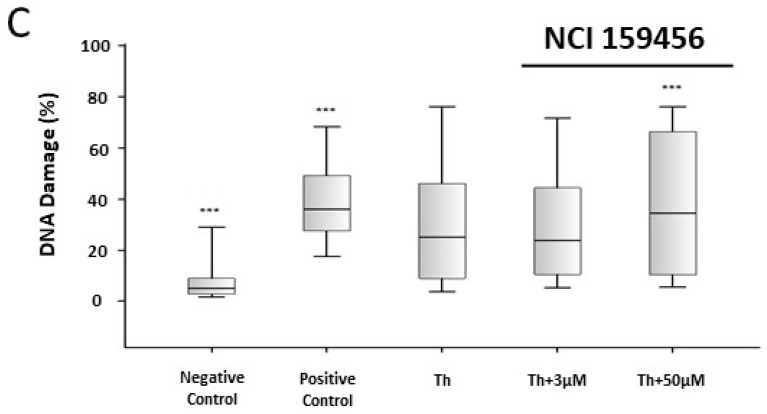
Analysis of genotoxicity in HPF (**A**) and A549 (**B**) cells exposed to the NCI 159456 PERK inhibitor and in A549 cells with Th-induced ER stress exposed to NCI 159456 compound (**C**), performed by the alkaline version of comet assay. All experiments were run in triplicate. The number of cells assessed in each experiment was 100. Box plots show median, first/third quartiles, and minimum/maximum values. *** *p* < 0.001 versus the negative control (**A**,**B**) and versus Th (**C**). Negative Control—untreated HPF (**A**) and A540 (**B**,**C**) cells; Positive Control—HPF (**A**) and A549 (**B**,**C**) cells treated with 5% dimethyl sulfoxide; 0.1% DMSO—HPF (**A**) and A549 (**B**) cells treated with the solvent, 0.1% dimethyl sulfoxide; Th—thapsigargin-treated A549 cells (ER-stressed A549).

The ACA was applied to assess the DNA damage induced by the NCI 159456 compound in HPF and A549 cell lines. The concentration of 0.1% DMSO and the investigated NCI 159456 PERK inhibitor did not cause significant DNA damage in HPF cells at any concentration after 24 h incubation compared to that in the negative control (Figure 5A). In contrast to HPF cells, the highest increase in DNA damage was observed in A549 cells treated with 50 μM of NCI 159456 PERK inhibitor compared to that in the negative control. The concentration of 0.1% DMSO did not evoke DNA damage in A549 cells (Figure 5B). Additionally, obtained results demonstrated significantly increased DNA damage in the ER-stressed A549 cells treated with 50 μM of NCI 159456 compound compared to that in the untreated ER-stressed A549 cells (Figure 5C).

### 3.4. Evaluation of the Level of Apoptosis

**Figure 6:**
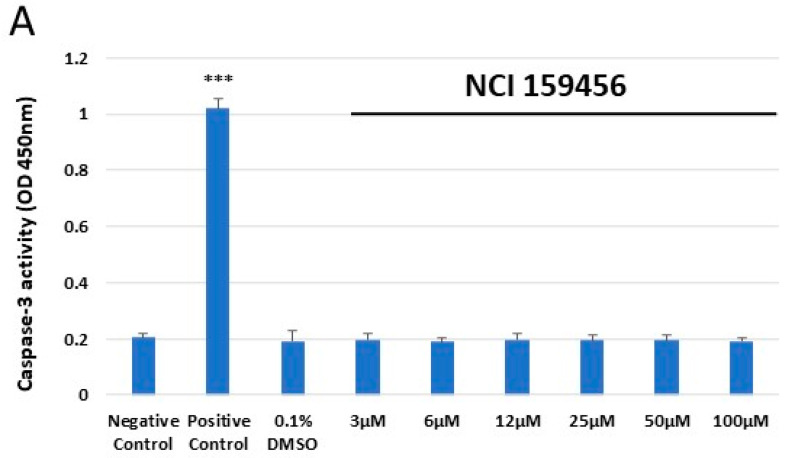

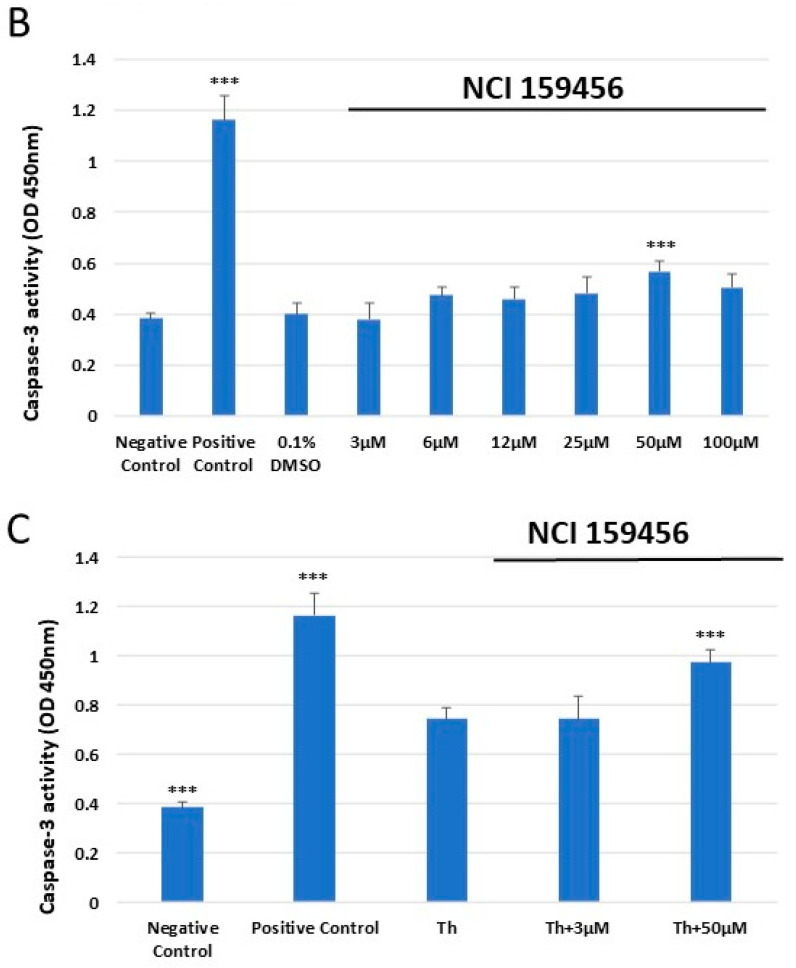
Evaluation of the level of apoptosis in HPF (**A**) and A549 (**B**) cells or ER-stressed A549 cells (**C**) after exposure to NCI 159456 PERK inhibitor. The assessment was performed by caspase-3 assay. All experiments were run in triplicate; values are expressed as mean ± SEM, n = 3. *** *p* < 0.001 versus the negative control (**A**,**B**) and versus Th (**C**). Negative Control—untreated HPF (**A**) and A549 (**B**,**C**) cells; Positive Control—HPF (**A**) and A549 (**B**,**C**) cells treated with 1 μM staurosporine; 0.1% DMSO—HPF (**A**) and A549 (**B**) cells treated with the solvent, 0.1% dimethyl sulfoxide; Th—thapsigargin-treated A549 cells (ER-stressed A549).

Colorimetric caspase-3 assay was used to assess the caspase-3 activity in HPF and A549 cell lines treated with the NCI 159456 PERK inhibitor. We noted a significant increase in the caspase-3 activity in HPF and A549 cells after treatment with 1 μM staurosporine for 16 h (Figure 6A–C). No significant induction of caspase-3-dependent apoptosis was observed after 24 h exposure of HPF to the NCI 159456 PERK inhibitor (3–50 μM) (Figure 6A). Furthermore, we demonstrated a relevant increase in caspase-3 activity in A549 cells incubated with 50 μM of NCI 159456 compound compared to that in the negative control (Figure 6B). Inhibitor solvent 0.1% DMSO did not induce caspase-3 activity both in HPF and A549 cell lines (Figure 6A,B). Additionally, the obtained results showed a significant increase in caspase-3 level in the ER-stressed A549 cells treated with 50 μM of NCI 159456 compound compared to that in the untreated ER-stressed A549 cells (Figure 6C).

### 3.5. Evaluation of the Level of Reactive Oxygen Species (ROS)

**Figure 7:**
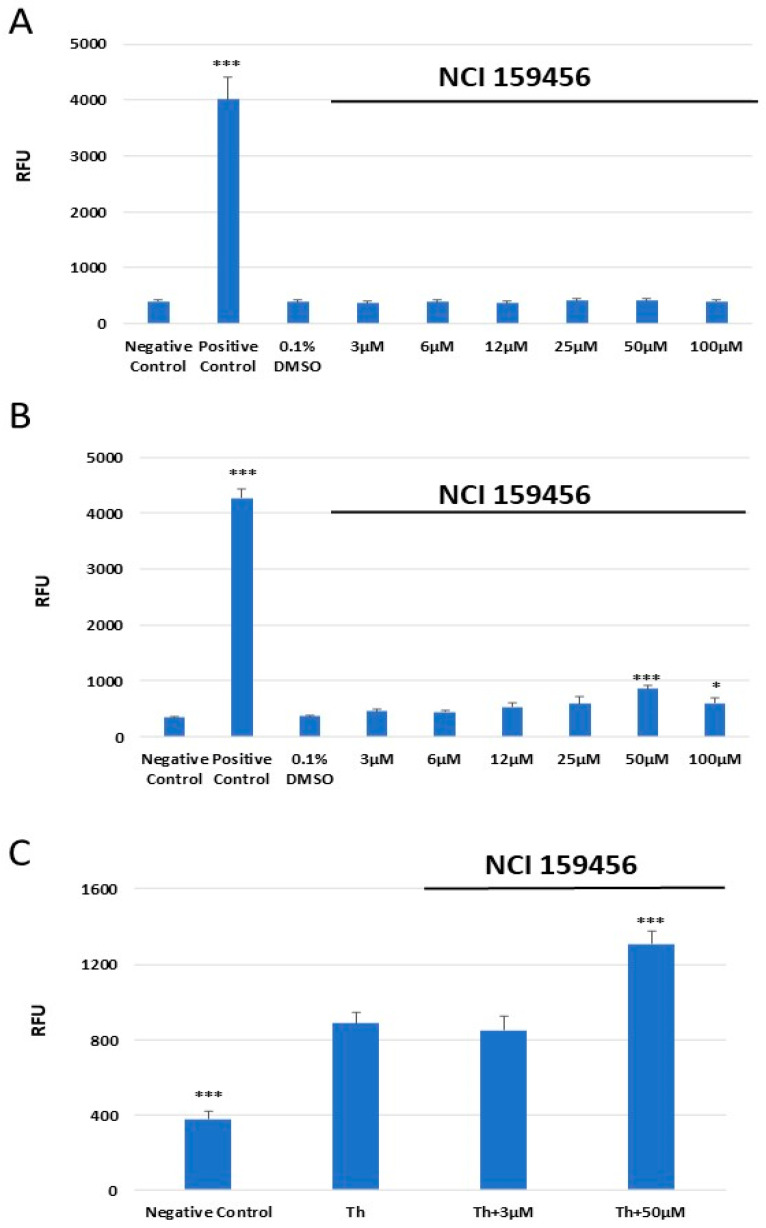
Analysis of the reactive oxygen species (ROS) generation in HPF (**A**) and A549 (**B**) cells or ER-stressed A549 cells (**C**) after treatment with NCI 159456 PERK inhibitor. The test was performed using the ROS Detection Assay Kit. All experiments were run in triplicate; values are expressed as mean ± SEM, n = 3. * *p* < 0.05, *** *p* < 0.001 versus the negative control (**A**,**B**) and versus Th (**C**). Negative Control—untreated HPF (**A**) and A549 (**B**,**C**) cells; Positive Control—HPF (**A**) and A549 (**B**) cells treated with ROS inducer; 0.1% DMSO—HPF (**A**) and A549 (**B**) cells treated with the solvent, 0.1% dimethyl sulfoxide; Th—thapsigargin-treated A549 cells (ER-stressed A549).

ROS Detection Assay Kit was applied to evaluate the level of ROS in HPF and A549 cells after exposure to the investigated NCI 159456 PERK inhibitor. There were no significant changes in the ROS level in HPF cells after treatment with the NCI 159456 PERK inhibitor at any concentration used and its solvent 0.1% DMSO compared with that in the negative control (Figure 7A). However, obtained results showed a significantly increased ROS level in A549 cells after their exposure to 50 μM of NCI 159456 compound compared to that in the negative control. We noted that 0.1% DMSO did not induce a significant increase in ROS level in A549 cells (Figure 7B). Furthermore, the obtained results demonstrated a significantly increased ROS level in the ER-stressed A549 cells treated with 50 μM of NCI 159456 compound compared to that in the untreated ER-stressed A549 cells (Figure 7C).

## 4. Discussion

Multiple internal and external stimuli, including cigarette smoke exposure, the leading causal factor of lung cancers, can induce chronic ER stress and UPR recruitment, which is a crucial pathway in tumorigenesis [23,24]. The upregulation of ER stress-related proteins was demonstrated to positively correlate with lymph node metastasis and poor prognosis in NSCLC [27]. Recent reports suggest a pleiotropic effect of PERK on carcinogenesis through the activation of numerous non-canonical signaling pathways [28,29]. It was established that the PERK-eIF2α-ATF4 pathway activation promotes angiogenesis, invasion, and epithelial–mesenchymal transition, thereby increasing the metastatic potential in various cancers, including NSCLC [30,31,32,33]. Significant enrichment of ATF4 target genes was detected in advanced lung adenocarcinomas with unfavorable prognosis, high risk of recurrence, and increased resistance to cisplatin [22,34]. The inhibition of eIF2α and ATF4 in nutrient-deficient conditions disrupted amino acid homeostasis in multiple stages and molecular subtypes of NSCLC, impairing tumor cell growth and motility [12,22]. Moreover, an acidic tumor microenvironment was demonstrated to induce ROS-mediated ER stress and upregulate ER stress-related proteins, such as p-PERK, p-eIF2α, DDIT3, spliced X-box binding protein 1 (XBP1s), and glucose-regulated protein 78 (GRP78), which promote both autophagy and cell survival, in NSCLC in vitro model [35,36]. Thus, to assess the biological effect of the investigated NCI 159456 inhibitor, our research was conducted on the A549 cell line treated with Th, which constituted an ER stress inducer.

The adaptive PERK/p-eIF2α branch of the UPR has been identified as a critical component of tumorigenesis, particularly in KRAS (Kirsten rat sarcoma viral oncogene homolog)-driven tumors representing an NSCLC subtype often resistant to treatment [23]. Therefore, in the present study, we evaluated the effectiveness of a pre-selected small-molecule PERK inhibitor on human lung carcinoma epithelial cells (A549)—a representative NSCLC cell line with mutations in the KRAS gene [37]. A normal human pulmonary fibroblasts (HPF) cell line was used as the control to assess the specificity of the tested PERK inhibitor and any potential adverse side effects of the inhibitor. Results obtained in this study have confirmed that the chosen inhibitor of the PERK-dependent UPR signaling pathway may constitute a targeted treatment strategy against NSCLC. Moreover, the fact that the tested compound had no significant effect on HPF demonstrates its specificity towards cancerous cells and the possibility of no severe side effects in healthy tissues.

Multiple PERK-specific inhibitors, such as GSK2606414, NCI 12487, and HC-5404, have recently been reported to display significant anti-tumor efficacy. These inhibitors could affect disease progression and prevent recurrence in diverse tumor models, including multiple myeloma, colorectal cancer, and renal tumor [18,19,20]. Intriguingly, some PERK-mediated UPR signaling inhibitors are currently being investigated in lung cancer models. The newest study on PERK inhibitor HC4 with single-cell gene expression profiling and imaging reported that a significant proportion of solitary disseminated cancer cells in the lungs were dormant and displayed PERK-dependent ER stress. Thus, PERK inhibitors could be applied as a novel strategy specifically affecting solitary disseminated cancer cells that originate metastases [17]. 

A study on A549 and H358 cells demonstrated that the inhibitors of PERK (GSK2656157), other UPR-related proteins, heat shock protein 90 (HSP90), and the receptor tyrosine kinase AXL can synergistically enhance the antitumor effects of pemetrexed and trametinib, suggesting a rational combination strategy to treat KRAS-mutant lung cancer. HSP90 was also shown to be essential for PERK/c-Jun N-terminal kinase/activating transcription factor 2 (PERK/JNK/ATF2)-dependent pro-survival response, and the inhibition of HSP90 leads to eIF2α/DDIT3-mediated apoptosis [38]. Another study exploring the role of ER stress and autophagy in cisplatin-induced apoptosis in NSCLC cells has shown that treatment with ER stress inhibitor 4-phenylbutyric acid or tauroursodeoxycholic acid sodium and an autophagic inhibitor 3-methyladenine or chloroquine was able to enhance cisplatin-induced apoptosis [39].

On the other hand, pazopanib, a multitargeted tyrosine kinase inhibitor, which is known to induce ROS-mediated ER stress and apoptosis in lung cancer, was evaluated in a phase III trial as a maintenance therapy after standard first-line platinum-based chemotherapy in patients with advanced NSCLC [40]. However, this study was stopped because of a lack of efficacy based on strict progression-free survival (PFS) criteria at a futility interim analysis [41]. Nifuroxazide was demonstrated to induce the apoptosis of H1299 NSCLC cells via the ROS/Ca^2+^/PERK-ATF4-DNA damage-inducible transcript 3 (CHOP) signaling pathway [42], and butein (3,4,2′,4′-tetrahydroxychalcone) was also reported to induced apoptosis and G2/M cell cycle arrest via the PERK/eIF2α/CHOP pathway in A549 and PC-9 NSCLC cell lines [43].

Intriguingly, metformin, an old anti-hyperglycemic drug that demonstrates numerous extra-metabolic actions, was recently observed to enhance the antitumor activity of MEK-I in human LKB1-wild-type NSCLC cell lines, independently of KRAS mutational status, through downregulation of the GLI Family Zinc Finger 1 (GLI1) and reduction of the nuclear factor kappa-light-chain-enhancer of activated B cells (NF-kB)-mediated transcription of matrix metalloproteinase-2 (MMP-2) and matrix metalloproteinase-9 (MMP-9) [44]. Its advantage of having mild and rare side effects makes it potentially valuable for oncology therapy in combination with antineoplastic drugs. 

Currently, available lung cancer treatment strategies, including diverse surgical methods, radiation-, chemo-, immuno-, and targeted therapy, present many disadvantages, such as poor bioavailability, high-dose requirements, adverse effects, narrow therapeutic index, development of multiple drug resistance, and non-specific targeting. Over the past two decades, advances in molecular profiling have translated into the successful application of numerous targeted therapies and immunotherapies in selected NSCLC patient populations. Nevertheless, an incomplete understanding of the molecular mechanisms underlying the progression of NSCLC limits the application of precision medicine, which could otherwise improve patients’ survival [45]. Tumor heterogeneity and drug resistance are significant difficulties in cancer medicine, especially in KRAS-NSCLC, which is particularly resistant to targeted therapy and first-line chemotherapy [38]. Despite substantial improvements in lung cancer treatment, the prognosis for NSCLC remains poor, with an overall survival (OS) of less than 9% in advanced NSCLC. Therefore, investigation of new molecular targets to overcome treatment resistance in NSCLC is warranted [46]. Hence, the present study offers further insight into the molecular mechanisms underlying NSCLC pathology and highlights the effectiveness of PERK inhibition against NSCLC cells in normal and ER stress conditions. Considering the specificity of the analyzed PERK inhibitor and its minimal impact on normal cells, NCI 159456 could become a potential treatment strategy against NSCLC to improve outcomes for NSCLC patients.

## 5. Conclusions

The present study investigated the biological effect of the selective PERK inhibitor NCI 159456 on NSCLC cells and pulmonary fibroblasts in vitro. Treatment of both normal and ER-stressed A549 cells with NCI at 50 µM significantly increased the mRNA expression level of pro-apoptotic genes (*ATF4*, *DDIT3*, *BAX*) and decreased the level of the anti-apoptotic *Bcl-2* gene. Moreover, 50 µM NCI 159456 significantly reduced NSCLC cell viability and increased the level of DNA damage both under normal and ER stress conditions. Further studies demonstrated that NCI 159456 at the indicated concentration significantly induced apoptosis and increased the ROS level in normal and ER-stressed A549 cells. At the same time, the inhibitor did not affect normal pulmonary cells HPF at any used concentration. The results obtained support the potential application of PERK inhibitors for targeted therapy against NSCLC.

## Data Availability

The data generated in the present study may be requested from the corresponding author.

## References

[1] Sung H., Ferlay J., Siegel R.L., Laversanne M., Soerjomataram I., Jemal A., Bray F. (2021). Global Cancer Statistics 2020: GLOBOCAN Estimates of Incidence and Mortality Worldwide for 36 Cancers in 185 Countries. CA Cancer J. Clin..

[2] Ganti A.K., Klein A.B., Cotarla I., Seal B., Chou E. (2021). Update of Incidence, Prevalence, Survival, and Initial Treatment in Patients with Non–Small Cell Lung Cancer in the US. JAMA Oncol..

[3] Chen Z., Fillmore C.M., Hammerman P.S., Kim C.F., Wong K.-K. (2014). Non-Small-Cell Lung Cancers: A Heterogeneous Set of Diseases. Nat. Rev. Cancer.

[4] Chen X., Cubillos-Ruiz J.R. (2021). Endoplasmic Reticulum Stress Signals in the Tumour and Its Microenvironment. Nat. Rev. Cancer.

[5] Wang M., Kaufman R.J. (2014). The Impact of the Endoplasmic Reticulum Protein-Folding Environment on Cancer Development. Nat. Rev. Cancer.

[6] Hetz C., Zhang K., Kaufman R.J. (2020). Mechanisms, Regulation and Functions of the Unfolded Protein Response. Nat. Rev. Mol. Cell Biol..

[7] Salaroglio I.C., Panada E., Moiso E., Buondonno I., Provero P., Rubinstein M., Kopecka J., Riganti C. (2017). PERK induces Resistance to Cell Death Elicited by Endoplasmic Reticulum Stress and Chemotherapy. Mol. Cancer.

[8] Bu Y., Diehl J.A. (2016). PERK Integrates Oncogenic Signaling and Cell Survival during Cancer Development. J. Cell. Physiol..

[9] Cubillos-Ruiz J.R., Bettigole S.E., Glimcher L.H. (2017). Tumorigenic and Immunosuppressive Effects of Endoplasmic Reticulum Stress in Cancer. Cell.

[10] Hart L.S., Cunningham J.T., Datta T., Dey S., Tameire F., Lehman S.L., Qiu B., Zhang H., Cerniglia G., Bi M. (2012). ER Stress–Mediated Autophagy Promotes Myc-Dependent Transformation and Tumor Growth. J. Clin. Investig..

[11] Zhang W., Neo S.P., Gunaratne J., Poulsen A., Boping L., Ong E.H., Sangthongpitag K., Pendharkar V., Hill J., Cohen S.M. (2015). Feedback Regulation on PTEN/AKT Pathway by the ER Stress Kinase PERK Mediated by Interaction with the Vault Complex. Cell. Signal.

[12] Du J., Liu H., Mao X., Qin Y., Fan C. (2021). ATF4 Promotes Lung Cancer Cell Proliferation and Invasion Partially through Regulating Wnt/β-Catenin Signaling. Int. J. Med. Sci..

[13] Feng Y.-X., Jin D.X., Sokol E.S., Reinhardt F., Miller D.H., Gupta P.B. (2017). Cancer-Specific PERK Signaling Drives Invasion and Metastasis through CREB3L1. Nat. Commun..

[14] Shi Z., Yu X., Yuan M., Lv W., Feng T., Bai R., Zhong H. (2019). Activation of the PERK-ATF4 Pathway Promotes Chemo-Resistance in Colon Cancer Cells. Sci. Rep..

[15] Qiao Q., Sun C., Han C., Han N., Zhang M., Li G. (2017). Endoplasmic Reticulum Stress Pathway PERK-EIF2α Confers Radioresistance in Oropharyngeal Carcinoma by Activating NF-ΚB. Cancer Sci..

[16] Siwecka N., Rozpędek W., Pytel D., Wawrzynkiewicz A., Dziki A., Dziki Ł., Diehl J.A., Majsterek I. (2019). Dual Role of Endoplasmic Reticulum Stress-Mediated Unfolded Protein Response Signaling Pathway in Carcinogenesis. Int. J. Mol. Sci..

[17] Calvo V., Zheng W., Adam-Artigues A., Staschke K.A., Huang X., Cheung J.F., Nobre A.R., Fujisawa S., Liu D., Fumagalli M. (2023). A PERK-Specific Inhibitor Blocks Metastatic Progression by Limiting Integrated Stress Response–Dependent Survival of Quiescent Cancer Cells. Clin. Cancer Res..

[18] Bagratuni T., Patseas D., Mavrianou-Koutsoukou N., Liacos C.I., Sklirou A.D., Rousakis P., Gavriatopoulou M., Terpos E., Tsitsilonis O.E., Trougakos I.P. (2020). Characterization of a PERK Kinase Inhibitor with Anti-Myeloma Activity. Cancers.

[19] Rozpedek-Kaminska W., Piotrzkowska D., Galita G., Pytel D., Kucharska E., Dziki Ł., Dziki A., Majsterek I. (2022). Small-Molecule Inhibitors of the PERK-Mediated Unfolded Protein Response Signaling Pathway in Targeted Therapy for Colorectal Cancer. Pol. J. Surg..

[20] Stokes M.E., Calvo V., Fujisawa S., Dudgeon C., Huang S., Ballal N., Shen L., Gasparek J., Betzenhauser M., Taylor S.J. (2023). PERK Inhibition by HC-5404 Sensitizes Renal Cell Carcinoma Tumor Models to Antiangiogenic Tyrosine Kinase Inhibitors. Clin. Cancer Res..

[21] Xiao W., Sun Y., Xu J., Zhang N., Dong L. (2022). UORF-Mediated Translational Regulation of ATF4 Serves as an Evolutionarily Conserved Mechanism Contributing to Non-Small-Cell Lung Cancer (NSCLC) and Stress Response. J. Mol. Evol..

[22] Albert A.E., Adua S.J., Cai W.L., Arnal-Estapé A., Cline G.W., Liu Z., Zhao M., Cao P.D., Mariappan M., Nguyen D.X. (2019). Adaptive Protein Translation by the Integrated Stress Response Maintains the Proliferative and Migratory Capacity of Lung Adenocarcinoma Cells. Mol. Cancer Res..

[23] Ghaddar N., Wang S., Woodvine B., Krishnamoorthy J., van Hoef V., Darini C., Kazimierczak U., Ah-son N., Popper H., Johnson M. (2021). The Integrated Stress Response Is Tumorigenic and Constitutes a Therapeutic Liability in KRAS-Driven Lung Cancer. Nat. Commun..

[24] Jorgensen E., Stinson A., Shan L., Yang J., Gietl D., Albino A.P. (2008). Cigarette Smoke Induces Endoplasmic Reticulum Stress and the Unfolded Protein Response in Normal and Malignant Human Lung Cells. BMC Cancer.

[25] Emanuelli G., Nassehzadeh-Tabriz N., Morrell N.W., Marciniak S.J. (2020). The Integrated Stress Response in Pulmonary Disease. Eur. Respir. Rev..

[26] Golubovskaya V.M., Palma N.L., Zheng M., Ho B., Magis A., Ostrov D., Cance W.G. (2013). A Small-Molecule Inhibitor, 5′-O-Tritylthymidine, Targets FAK and Mdm-2 Interaction, and Blocks Breast and Colon Tumorigenesis in vivo. Anticancer. Agents Med. Chem..

[27] Xu Y., Chen Z., Zhang G., Xi Y., Sun R., Wang X., Wang W., Chai F., Li X. (2016). HSP90B1 Overexpression Predicts Poor Prognosis in NSCLC Patients. Tumor Biol..

[28] Del Vecchio C.A., Feng Y., Sokol E.S., Tillman E.J., Sanduja S., Reinhardt F., Gupta P.B. (2014). De-Differentiation Confers Multidrug Resistance Via Noncanonical PERK-Nrf2 Signaling. PLoS Biol..

[29] Peñaranda-Fajardo N.M., Meijer C., Liang Y., Dijkstra B.M., Aguirre-Gamboa R., den Dunnen W.F.A., Kruyt F.A.E. (2019). ER Stress and UPR Activation in Glioblastoma: Identification of a Noncanonical PERK Mechanism Regulating GBM Stem Cells through SOX2 Modulation. Cell Death Dis..

[30] Wang Y., Alam G.N., Ning Y., Visioli F., Dong Z., Nör J.E., Polverini P.J. (2012). The Unfolded Protein Response Induces the Angiogenic Switch in Human Tumor Cells through the PERK/ATF4 Pathway. Cancer Res..

[31] Feng Y., Sokol E.S., Del Vecchio C.A., Sanduja S., Claessen J.H.L., Proia T.A., Jin D.X., Reinhardt F., Ploegh H.L., Wang Q. (2014). Epithelial-to-Mesenchymal Transition Activates PERK–EIF2α and Sensitizes Cells to Endoplasmic Reticulum Stress. Cancer Discov..

[32] Dey S., Sayers C.M., Verginadis I.I., Lehman S.L., Cheng Y., Cerniglia G.J., Tuttle S.W., Feldman M.D., Zhang P.J.L., Fuchs S.Y. (2015). ATF4-Dependent Induction of Heme Oxygenase 1 Prevents Anoikis and Promotes Metastasis. J. Clin. Investig..

[33] Wang L., Wen J., Sun Y., Yang X., Ma Y., Tian X. (2023). Knockdown of NUPR1 Inhibits Angiogenesis in Lung Cancer through IRE1/XBP1 and PERK/EIF2α/ATF4 Signaling Pathways. Open Med..

[34] Tanabe M., Izumi H., Ise T., Higuchi S., Yamori T., Yasumoto K., Kohno K. (2003). Activating Transcription Factor 4 Increases the Cisplatin Resistance of Human Cancer Cell Lines. Cancer Res..

[35] Seong Y.-A., Shin P.-G., Yoon J.-S., Yadunandam A.K., Kim G.-D. (2014). Induction of the Endoplasmic Reticulum Stress and Autophagy in Human Lung Carcinoma A549 Cells by Anacardic Acid. Cell Biochem. Biophys..

[36] Xie W.-Y., Zhou X.-D., Li Q., Chen L.-X., Ran D.-H. (2015). Acid-Induced Autophagy Protects Human Lung Cancer Cells from Apoptosis by Activating ER Stress. Exp. Cell Res..

[37] Gwinn D.M., Lee A.G., Briones-Martin-del-Campo M., Conn C.S., Simpson D.R., Scott A.I., Le A., Cowan T.M., Ruggero D., Sweet-Cordero E.A. (2018). Oncogenic KRAS Regulates Amino Acid Homeostasis and Asparagine Biosynthesis via ATF4 and Alters Sensitivity to L-Asparaginase. Cancer Cell.

[38] Yang H., Liang S.-Q., Xu D., Yang Z., Marti T.M., Gao Y., Kocher G.J., Zhao H., Schmid R.A., Peng R.-W. (2019). HSP90/AXL/EIF4E-Regulated Unfolded Protein Response as an Acquired Vulnerability in Drug-Resistant KRAS-Mutant Lung Cancer. Oncogenesis.

[39] Shi S., Tan P., Yan B., Gao R., Zhao J., Wang J., Guo J., Li N., Ma Z. (2016). ER Stress and Autophagy Are Involved in the Apoptosis Induced by Cisplatin in Human Lung Cancer Cells. Oncol. Rep..

[40] Li Y., Chen C., Liu H., Li C., Zhang Z., Wang C. (2022). Pazopanib Restricts Small Cell Lung Cancer Proliferation via Reactive Oxygen species-mediated Endoplasmic Reticulum Stress. Thorac. Cancer.

[41] O’Brien M.E.R., Gaafar R., Hasan B., Menis J., Cufer T., Popat S., Woll P.J., Surmont V., Georgoulias V., Montes A. (2015). Maintenance Pazopanib versus Placebo in Non-Small Cell Lung Cancer Patients Non-Progressive after First Line Chemotherapy: A Double Blind Randomised Phase III Study of the Lung Cancer Group, EORTC 08092 (EudraCT: 2010-018566-23, NCT01208064). Eur. J. Cancer.

[42] Li D., Liu L., Li F., Ma C., Ge K. (2023). Nifuroxazide Induces the Apoptosis of Human Non-small Cell Lung Cancer Cells through the Endoplasmic Reticulum Stress PERK Signaling Pathway. Oncol. Lett..

[43] Di S., Fan C., Ma Z., Li M., Guo K., Han D., Li X., Mu D., Yan X. (2019). PERK/EIF-2α/CHOP Pathway Dependent ROS Generation Mediates Butein-Induced Non-Small-Cell Lung Cancer Apoptosis and G2/M Phase Arrest. Int. J. Biol. Sci..

[44] Della Corte C.M., Ciaramella V., Di Mauro C., Castellone M.D., Papaccio F., Fasano M., Sasso F.C., Martinelli E., Troiani T., De Vita F. (2016). Metformin Increases Antitumor Activity of MEK Inhibitors through GLI1 Downregulation in LKB1 Positive Human NSCLC Cancer Cells. Oncotarget.

[45] Herbst R.S., Morgensztern D., Boshoff C. (2018). The Biology and Management of Non-Small Cell Lung Cancer. Nature.

[46] Araghi M., Mannani R., Heidarnejad Maleki A., Hamidi A., Rostami S., Safa S.H., Faramarzi F., Khorasani S., Alimohammadi M., Tahmasebi S. (2023). Recent Advances in Non-Small Cell Lung Cancer Targeted Therapy; an Update Review. Cancer Cell Int..

